# Commentary: Discriminating α-synuclein strains in parkinson's disease and multiple system atrophy

**DOI:** 10.3389/fnins.2020.00802

**Published:** 2020-08-25

**Authors:** Lisa Fellner, Kurt A. Jellinger, Gregor K. Wenning, Johannes Haybaeck

**Affiliations:** ^1^Division of Neurobiology, Department of Neurology, Medical University of Innsbruck, Innsbruck, Austria; ^2^Institute of Clinical Neurobiology, Vienna, Austria; ^3^Department of Pathology, Neuropathology and Molecular Pathology, Medical University of Innsbruck, Innsbruck, Austria; ^4^Department of Neuropathology, Diagnostic & Research Institute of Pathology, Medical University of Graz, Graz, Austria

**Keywords:** Parkinson's disease, multiple system atrophy (MSA), alpha-synuclein, protein misfolding cyclic amplification (PMCA), neurodegenerative disease

## Introduction

Distinguishing between the two α-synucleinopathies, Parkinson's disease (PD), and multiple system atrophy (MSA), is difficult, especially early in the disease, as PD and MSA share many clinical features (Fanciulli and Wenning, 2015). Early diagnosis would have a tremendous impact on treatment options. Hence, the development of a reliable marker has been pursued with much effort. Body fluids, including blood and cerebrospinal fluid (CSF), provide access to a range of biomarkers. Inflammatory markers, such as IL-6, TNF-α (Starhof et al., 2018), and α-synuclein (AS) concentrations, have been investigated extensively (Aerts et al., 2012), yet no differentiation between PD and MSA is possible to date. Neurofilament light chain (NLF) was also investigated and was found to be elevated in the CSF of MSA patients compared to that in PD patients (Singer et al., 2020).

Shahnawaz and colleagues established a method, the AS protein misfolding cyclic amplification (PMCA) technology, allowing the detection of very small concentrations of AS aggregates in CSF (Shahnawaz et al., 2020). Using this test, they were able to differentiate between AS aggregates from PD and MSA patients. Furthermore, they demonstrated that AS aggregates from PD patients have a different signature compared to those from MSA patients and thus correspond to different conformational strains. AS from the CSF of PD patients revealed different biochemical and structural properties compared to AS from the CSF of patients with MSA. The biochemical and the structural signatures of AS aggregates were maintained after serial replication using AS-PMCA. Furthermore, the authors could show that even low amounts of AS derived from MSA patients are highly cytotoxic, whereas much higher amounts of AS derived from PD patients are needed to induce cytotoxic effects.

## How Sensitive and Specific is PMCA for the Distinction of PD From MSA?

This study is an important step toward the early distinction of PD from MSA based on easily accessible biomarkers. However, further work is required to determine the approach's sensitivity and specificity. In particular, not all CSF samples from MSA and PD patients showed the typical AS signature of MSA or PD using AS-PMCA. Some exhibited the aggregation profile of MSA (three samples), although these were PD cases and *vice versa* (four samples). Furthermore, not all analyzed CSF samples from PD or MSA patients exhibited AS aggregates using AS-PMCA (10 samples from patients with MSA and six samples from patients with PD). It would be interesting to know if these patient samples were also negative for AS aggregates using a different test. The overall sensitivity of the AS-PMCA test was evaluated, excluding the samples that were not detected correctly, achieving 95.4% (146 correct detected samples out of 153). However, if one would include all samples (169) of all patients that were analyzed (also the above-mentioned samples that did not exhibit AS aggregates at all, *n* = 16), sensitivity reaches 86.4% (146 out of 169). If clinicians use solely this test to distinguish between PD and MSA at an early stage, there is still a chance that patients are not properly diagnosed. An early and accurate diagnosis would be of great importance to secure the correct treatment of patients.

The CSF samples of PD and MSA patients used in this study are not confirmed cases as a neuropathological confirmation would be necessary (Trojanowski et al., 2007; Gilman et al., 2008). In two recent brain bank studies, among patients diagnosed with MSA during life, only 62 and 79%, respectively, met the neuropathological criteria (Koga et al., 2015; Miki et al., 2019); a positive predictive diagnosis of MSA even in later disease stages was only from 60 to 90% (Osaki et al., 2009; Stankovic et al., 2019). The clinical diagnosis of probable PD or MSA in this study might not be accurate. Therefore, the sensitivity of the AS-PMCA might not be precise as well and would reduce the overall sensitivity of the test further.

## Discussion

Typically, human CSF collection is not a routine diagnostic procedure used for PD and MSA patients. It could be difficult to integrate this test into a routine workflow as it is also moderately invasive. The development of a blood-based AS-PMCA test would be good to incorporate into routine patient examination as a diagnostic tool. Furthermore, given that blood samples compared to CSF samples are easier to obtain, an improvement of the test sensitivity using blood samples might be a simpler approach as more patients could be included. However, it is clear that, before moving to blood samples, the first step had to go *via* CSF for confirming that the technology works.

Shahnawaz and colleagues developed a new method for the analysis of AS aggregates in the CSF of PD and MSA patients. The current study could pave the way toward improved early diagnosis based on blood samples. Moreover, a combined analysis of NLF and AS in CSF might increase the sensitivity of the test and could provide a better discrimination of MSA and PD. Different conformational AS strains in PD and MSA CSF were identified, which could facilitate the discrimination between the two diseases. However, some research questions remain. Is the test sensitive enough to identify PD and MSA patients at the early stages of the disease? Only discrimination of PD and MSA at a very early disease stage will be helpful to clinicians so they can choose the ideal treatment option. Further studies will be needed to examine the true sensitivity and the specificity of the AS-PMCA test and to determine its usefulness as a diagnostic tool for clinical routine diagnostics.

## Author Contributions

LF, KJ, GW, and JH were involved in the conception, drafting, and revisions of the manuscript. All authors contributed to the article and approved the submitted version.

## Conflict of Interest

The authors declare that the research was conducted in the absence of any commercial or financial relationships that could be construed as a potential conflict of interest.
